# The Heart-Brain Bypass: Paradoxical Septic Embolism Through a Patent Foramen Ovale (PFO)

**DOI:** 10.7759/cureus.94546

**Published:** 2025-10-14

**Authors:** Carlos Fagundo, Cooper Dodd, Butros Bazo

**Affiliations:** 1 Internal Medicine, BayCare Health System, Tampa, USA

**Keywords:** brain abscess, intra-arterial shunt, patent foramen ovale (pfo), right atrial cardiac mass, septic emboli

## Abstract

Brain abscesses are rare sequelae of paradoxical septic embolism from cardiac sources, particularly in the absence of valvular endocarditis. Diagnosis remains challenging, and a high index of clinical suspicion is often required. We present a 65-year-old female with a past medical history of hypertension and dyslipidemia with a chief complaint of altered mental status. She was found to have left-sided focal seizures and dysarthria. Computed tomography (CT) of the head with and without contrast, with subsequent confirmatory magnetic resonance imaging (MRI) of the head with contrast, revealed a 3.5 cm ring-enhancing lesion in the right frontal lobe, consistent with a brain abscess. Craniotomy and drainage yielded *Streptococcus intermedius*, suggesting a likely dental source. A transesophageal echocardiogram (TEE) identified a large multilobular right atrial mass and an interatrial shunt, likely a patent foramen ovale (PFO), enabling paradoxical embolization. AngioVac (AngioDynamics, Latham, NY, USA) aspiration of the right atrial mass, targeted antibiotic therapy, and guideline-directed medical therapy (GDMT) provided a multimodal treatment plan to best suit the patient’s individual needs.

This case illustrates the importance of considering atypical sources in brain abscesses of unclear origin. The necroinflammatory infiltrate found on pathology of the right atrial mass, in addition to abscess cultures demonstrating heavy growth of *S. intermedius*, suggests that the right atrial mass served as a nidus for septic embolization to the brain through the patient’s PFO. Due to a critical illness on initial presentation, the patient was started on empiric antibiotics on admission. Blood cultures and cultures of the right atrial mass were subsequently negative for any organisms. It was noted by clinical history that the patient had invasive dental work approximately two weeks prior to presentation. Given the above information, the most likely cause of the patient’s brain abscess was culture-negative nonvalvular bacterial endocarditis. In the absence of a mechanical valve and intravenous drug use, turbulent flow induced endothelial injury, likely exposing fibrin on the lateral wall of the right atrium. The patient was ultimately discharged with close outpatient follow-up with marked improvement in symptoms. Early recognition, advanced imaging, and multidisciplinary management, including neurosurgical intervention, cardiothoracic surgery, and infectious disease consultation, were critical for optimizing outcomes.

## Introduction

Brain abscesses are an uncommon source of intracranial infections [1]. Abscesses can arise from local infections in the head and neck, or from hematogenous spread [2-4]. Brain abscesses can also occur after neurosurgical procedures or trauma [4]. Management involves neurosurgical intervention for abscesses larger than 2.5 cm, and intravenous antibiotic therapy based on culture results [5,6]. The association between brain abscess and patent foramen ovale (PFO) remains poorly understood, but emerging evidence suggests that paradoxical septic embolization through a PFO may contribute to brain abscess formation [7]. A PFO can lead to brain abscesses by bypassing pulmonary filtration, allowing for brain abscess formation. This report underscores the importance of considering atypical cardiac lesions and paradoxical embolization in the differential diagnosis of cryptogenic brain abscesses.

## Case presentation

A 65-year-old immunocompetent woman with a history of hypertension, dyslipidemia, and no previously known cardiac disease presented to the emergency department with altered mental status, slurred speech, left upper extremity weakness with twitching, and confusion. Her family reported several days of progressive weakness and behavioral changes. On arrival, she was afebrile with blood pressure 150/70 mmHg and sinus tachycardia on electrocardiogram (EKG). Laboratory findings were notable for leukocytosis 14.2 th/uL (normal: 4.5th/uL-11.0 th/uL). Neurologic examination revealed right gaze preference, dysarthria, left-sided neglect with hemiparesis, and focal seizure activity involving the left upper extremity. Cardiopulmonary examination was unremarkable, with normal heart sounds, no murmurs, rubs, or gallops, and clear breath sounds bilaterally without wheezes, rales, or rhonchi.

**Figure 1:**
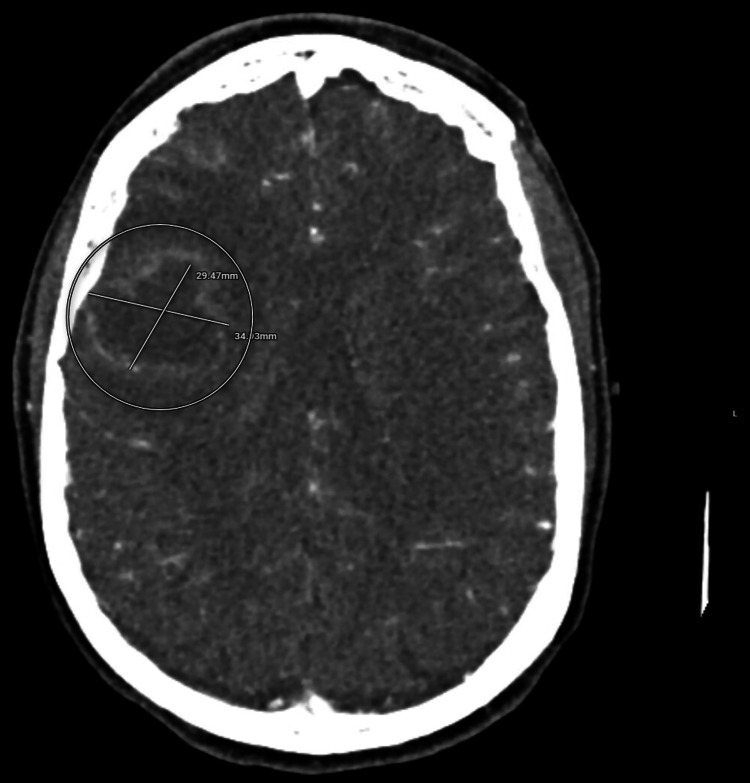
Computed tomography (CT) head with contrast, axial view, showing a 3.5 × 3.0 cm rim-enhancing mass in the right frontal region, best measured on the contrast-enhanced images.

Due to the patient’s initial presentation, a stroke alert was called to rule out any emergent conditions. Initial head computed tomography (CT) demonstrated a 3.5 × 3.0 cm rim-enhancing right frontal lobe mass with surrounding vasogenic edema and mild midline shift, but was otherwise negative for stroke (Figure 1). During her course in the emergency room, CT chest, abdomen, and pelvis were unremarkable for malignancy, though incidental hepatic cysts and vertebral compression fractures were noted.

**Figure 2:**
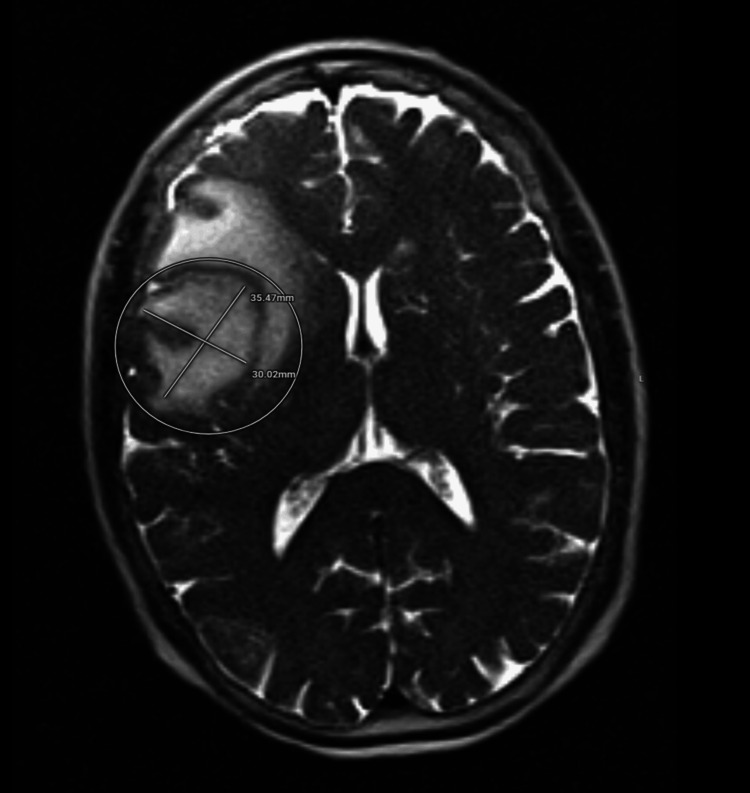
Brain magnetic resonance imaging (MRI), T2-weighted axial image, showing a rim-enhancing mass in the right frontal lobe measuring approximately 3.5 × 3.0 cm. Imaging features include central diffusion restriction within a T2 hypointense rim, most characteristic of a brain abscess, with marked surrounding vasogenic edema.

The patient was admitted for further evaluation, and a brain magnetic resonance imaging (MRI) was obtained shown in Figure 2, which showed a right frontal brain abscess. Infectious disease was consulted and started the patient on broad-spectrum antibiotics with meropenem and vancomycin (33.3 mg/kg intravenous three times daily and 16.7 mg/kg twice daily, respectively). Blood cultures were obtained prior to antibiotic administration, which did not show any growth. At that time, the patient was started on levetiracetam (1000 mg intravenous twice daily) for empiric seizure prophylaxis. Neurosurgery was consulted to evaluate a brain abscess, and recommended craniotomy with duraplasty plus drainage of the right frontal abscess. The patient tolerated the procedure well with no complications. Her postoperative neurologic status improved steadily, with recovery of alertness, orientation, and cooperation. Repeat head CT demonstrated no recurrent abscess formation. Cultures of the abscess fluid grew *Streptococcus intermedius*, consistent with an oral or dental source.

The patient’s cardiac evaluation revealed sinus tachycardia with prolonged QT interval, and a transthoracic echocardiogram (TTE) showed an ejection fraction (EF) of 30-35% with global hypokinesis and mild diastolic dysfunction, but no valvular vegetations. She was started on furosemide and bisoprolol (0.042 mg/kg twice daily and 0.67 mg/kg twice daily, respectively) in the setting of a new onset heart failure with reduced EF, with a wearable defibrillator for sudden cardiac death (SCD) prophylaxis in the setting of a critically reduced EF.

**Figure 3:**
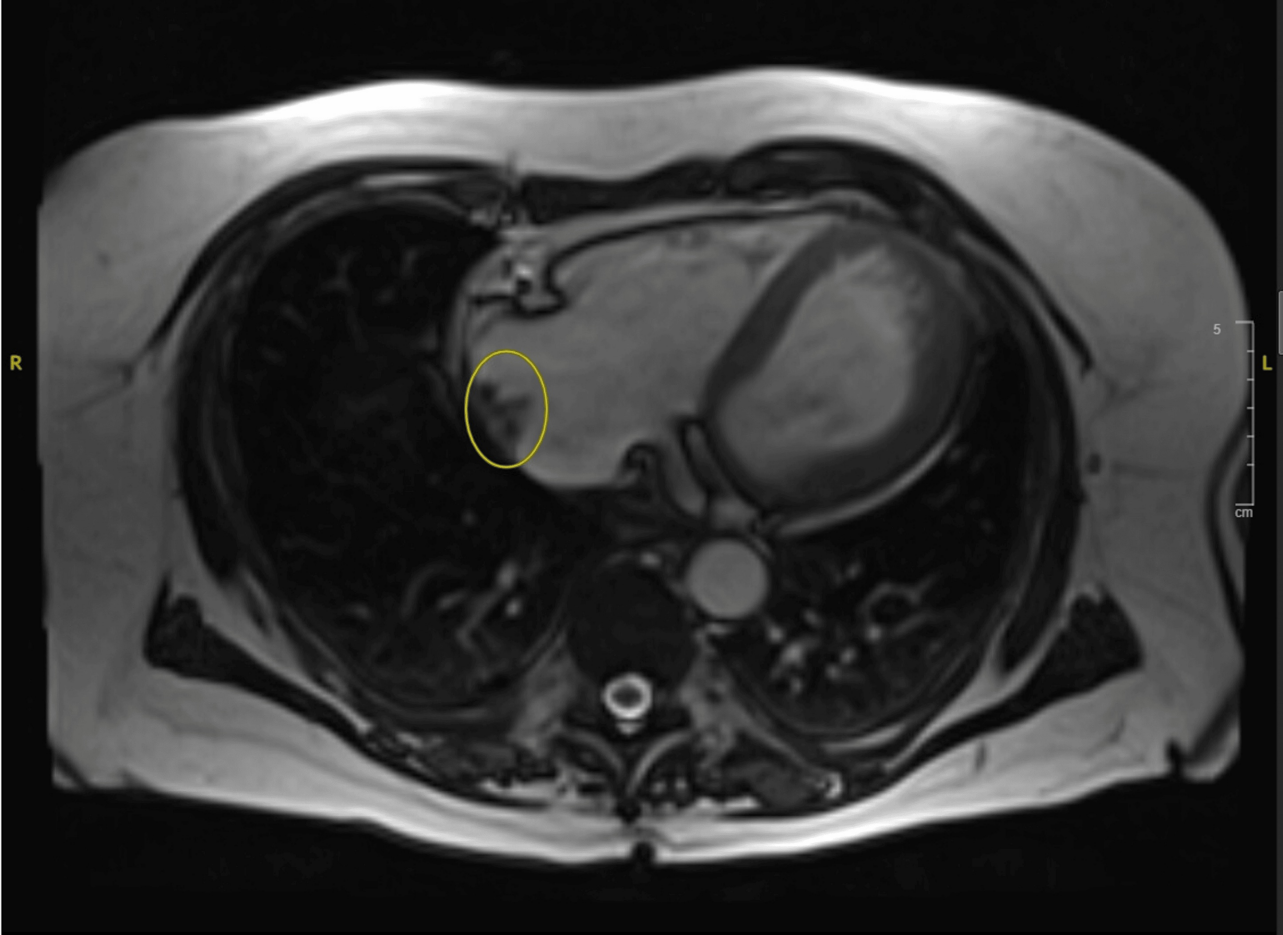
Cardiac magnetic resonance imaging (CMRI), T2-weighted axial view, showing a right atrial mass on the lateral wall measuring 1.9 × 1.2 cm. The right atrium is mildly enlarged. First-pass contrast imaging was not obtained due to technical issues, and no obvious delayed enhancement was seen in the lesion.

Further cardiac evaluation with a transesophageal echocardiogram (TEE) was done to rule out endocarditis. TEE revealed a large multilobular right atrial mass as well as an interatrial shunt, likely a PFO. No valvular vegetations were identified. Cardiothoracic surgery recommended AngioVac aspiration (AngioDynamics, Latham, NY, USA) of the right atrial mass. The procedure successfully aspirated multiple fragments of the atrial mass. Cultures from the right atrial mass were negative. Postprocedurally, the patient underwent cardiac magnetic resonance imaging (CMRI) showing a right atrial mass measuring 1.9 x 1.2 cm as seen in Figure 3. The patient was continued on guideline-directed medical therapy (GDMT) and was found to be clinically euvolemic.

She remained hemodynamically stable as she engaged in rehabilitation efforts with physical therapy. A 28-day course of intravenous meropenem (33.3 mg/kg intravenous three times daily) was continued on discharge. On outpatient follow-up with cardiology, her echocardiography demonstrated no residual mass, EF improvement to 35-39%. Further follow-up was arranged with cardiothoracic surgery and neurosurgery.

## Discussion

**Figure 4:**
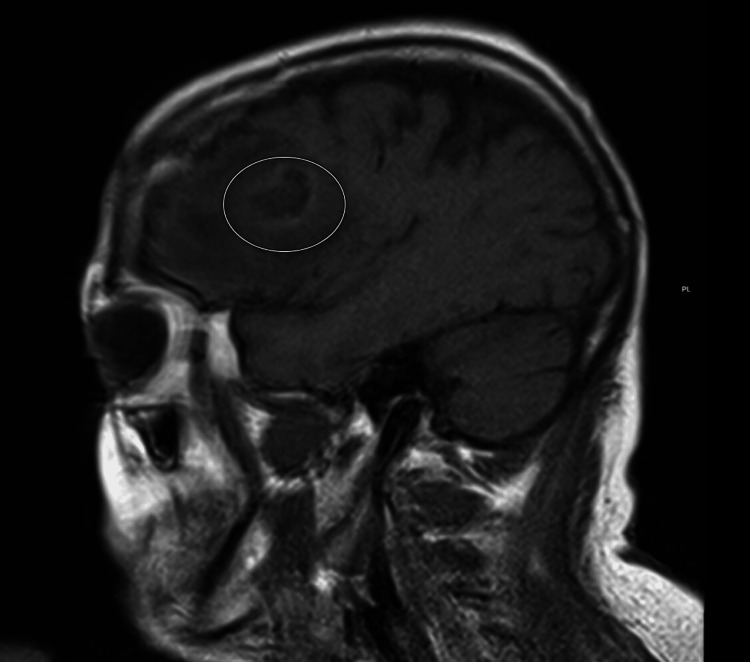
T1-weighted sagittal magnetic resonance imaging (MRI) of the head with contrast, showing a rim-enhancing mass in the right frontal lobe without involvement of the maxillary sinus.

This case illustrates the unusual presentation of a brain abscess in association with a right atrial mass and an interatrial communication. Brain abscesses are uncommon in developed countries, with an incidence of 0.3-1.3 per 100,000 population per year, but they carry significant morbidity and mortality. Despite advances in imaging, neurosurgical techniques, and antimicrobial therapy, diagnosis and treatment remain challenging [1,2]. The most common etiologies include contiguous spread from sinusitis, otitis media, or odontogenic infections, as well as hematogenous dissemination from endocarditis or pulmonary infections [3,4]. The presence of *S. intermedius* and a PFO supports a hematogenous mechanism of paradoxical embolization. Brain abscess formation can arise from odontogenic infection through the maxillary sinus, which cannot be entirely excluded. However, imaging did not reveal maxillary sinus involvement (Figure 4), and no intraoperative findings supported direct continuity.

Therefore, while hematogenous seeding via the PFO remains the most plausible primary pathway, a localized odontogenic source should be considered in the evaluation of patients with brain abscesses. In our patient, the identification of *S. intermedius*, a member of the *Streptococcus anginosus* group, has a propensity for abscess formation. This favors an odontogenic source infection [8,9]. While cultures from the right atrial mass were negative, we attribute this to prior administration of antibiotics before the AngioVac aspiration procedure, which may have sterilized the specimen.

The cardiac findings in this case were particularly notable. The patient had no prior structural heart disease, yet transthoracic and transesophageal echocardiography revealed dilated cardiomyopathy with reduced EF and a large multilobular right atrial mass. Right-sided intracardiac masses are relatively rare in the absence of intravenous drug use, indwelling catheters, or prosthetic material [10]. The AngioVac aspiration system provided a minimally invasive means of debulking the infected thrombus, avoiding the higher risks associated with open cardiac surgery in a patient who had recently undergone craniotomy [11].

The concomitant presence of a PFO had important pathophysiologic implications. Normally, septic emboli originating in the right heart are filtered in the pulmonary circulation, leading to septic pulmonary emboli or lung abscesses [12]. In this case, the interatrial shunt created a pathway for paradoxical embolization directly into the cerebral circulation. While PFOs are relatively common in the general population, present in up to 25% of adults, their role as conduits for embolic stroke and brain abscess formation has been increasingly recognized [13,14]. Management of brain abscesses requires prompt diagnosis, surgical intervention when feasible, and prolonged intravenous antimicrobial therapy. The PFO-Associated Stroke Causal Likelihood (PASCAL) classification and the Risk of Paradoxical Embolism (RoPE) score have been proposed to help stratify the causal association and recurrence risk in patients with PFO-related embolic events [15,16]. Although primarily validated in the context of ischemic stroke, these frameworks underscore the clinical relevance of PFO as a conduit for paradoxical emboli and provide context for the unusual presentation of brain abscess in this case.

The Infectious Disease Society of America (IDSA) develops guidelines using a panel of experts that performs systematic reviews of available evidence using the grading of recommendations, assessment, development, and evaluation (GRADE) system. Recommendations for clinical practice are provided with a strength of recommendation (A, B, or C) as well as the quality of evidence (I, II, or III). IDSA guidelines recommend stereotactic aspiration or craniotomy with excision for abscesses (IIA) >2.5 cm or associated with mass effect, in combination with empiric broad-spectrum antibiotics tailored once culture results are available [5]. In this case, the patient underwent neurosurgical drainage of the right frontal abscess with subsequent resolution of mass effect. Cultures grew *S. intermedius*, and she was treated with a six-week course of intravenous meropenem (33.3 mg/kg intravenous meropenem three times daily), in accordance with guideline recommendations for streptococcal abscesses [5,6]. Seizure prophylaxis with levetiracetam (20 mg/kg intravenous two times daily) was also initiated, given that seizures are a common sequelae of brain abscess [17].

One may consider unusual cardiac lesions and potential right-to-left shunts as sources for paradoxical embolization in future patients presenting with cryptogenic brain abscess.

## Conclusions

This case underscores the importance of multidisciplinary management. The interplay between neurosurgery, infectious disease, cardiology, and cardiothoracic surgery was critical in achieving a favorable outcome. More broadly, it emphasizes that in patients with brain abscesses without an apparent source, occult cardiac pathology, including PFO and right-sided intracardiac masses, should be considered. Recognition of paradoxical embolization through a PFO may be lifesaving, as it changes both diagnostic evaluation and management strategy.
